# Book Reviews

**Published:** 1823-12

**Authors:** 


### CRITICAL ANALYSIS

OF

### ENGLISH AND FOREIGN LITERATURE

RELATIVE TO THE VARIOUS BRANCHES OF

### Medical Science.

Quæ laudanda forent, et quæ culpanda, vicissim

Illa, prius, cretâ; mox hæc, carbone, *notamus*.—PERSIUS.

### DIVISION I.

### ENGLISH.

ART. I.—*Lectures on the Operative Surgery of the Eye: being the Substance of that part of the Author's Course of Lectures on the Principles and Practice of Surgery which relates to the Diseases of that Organ: published for the purpose of assisting in bringing the Management of these Complaints within the Principles which regulate the Practice of Surgery in general.* By G. J. GUTHRIE, Deputy Inspector of Hospitals during the Peninsular War; Surgeon to the Royal Westminster Infirmary for Diseases of the Eye; Consulting Surgeon to the Western Dispensary for the Diseases of Women and Children; Assistant Surgeon to the Westminster Hospital; Lecturer on Surgery, &c. &c. &c.—8vo. pp. 523. Burgess and Hill, London. 1823.

THE limited space in which the reviewer is compelled to compress his account of the labours of his professional brethren, too often prevents his doing ample justice to their works, and leaves him only the humbler task of recording their general merits, and recommending their attentive perusal. In the instance at present before us, we feel ourselves in this situation. Mr. GUTHRIE's work occupies 500 pages, and is illustrated with three plates; it comprises a very ample and extended account of those diseases of the eye and its appendages which usually lead to an operation; and, being the substance of the Lectures which that gentleman delivers at the Royal Westminster Infirmary for Diseases of the Eyes, it contains, of course, a great deal of explanatory matter, and a longer history of most of those diseases than it would be necessary to detail in a work intended solely for the educated practitioner. This explanation must be our apology for offering but a meagre account of a volume replete with valuable matter, and which evinces great research, and an intimate acquaintance with the works of the most esteemed authors, both ancient and modern. In a short Preface, our author observes, that—

"Previously to the year 1817, no lectures were delivered in Great Britain on the diseases of the Eye, unless the concise observations which were made in the schools of anatomy on some of the operations be considered as such. The consequence was, that a knowledge of these complaints was not presumed to form a part of the education of a surgeon; and the public, in applying for relief to those gentlemen who made this branch of the profession their only occupation, encouraged rather than repressed a propensity to idleness, which so frequently induces mankind to neglect that which does not appear to be immediately necessary, or which is not directly required. The various casualties of a protracted war in distant climates, which rendered it imperative on many of the physicians and surgeons of the army and navy to pay greater attention to the diseases of the eye, together with the impulse given by the application and success of the late Mr. Saunders, gradually, however, began to cause some change in the ideas of medical men; and, at last, to convince many of the propriety of their acquiring a knowledge of these diseases by the same means, and in the same manner, as were found essentially necessary in the study of medicine and surgery in general."

This leads him to notice the institution of the Infirmary, where the lectures, of which this publication forms a part, were delivered; and he informs us, that they are published at the urgent desire of "a great body of students, who were solicitous to have an opportunity" of reviewing those opinions more at leisure, and of referring to them when they might stand in need of assistance. After combating some popular prejudices relative to the exquisite sensibility of the eye in a state of health, the contrary of which is known to be the case by every well-informed medical man, our author next adverts to the difficulties supposed to attend the performance of operations on the eye, and which he confutes; but we may be permitted here to observe that, however true this may be, we are inclined to think that the performance of operations on the eye must always be confined to a very few persons. In fact, operative surgery, in all its branches, is under the same predicament; for, however well any surgeon may understand the performance of an operation, still the public will, with a natural bias, apply only to those whose public situations afford them an extensive field of practice; and we shall find that the performance of lithotomy and other great operations is, in this town, in the hands of a very limited number of practitioners: so it will always be, we apprehend, with regard to the eye; but a knowledge of the diseases of that organ, and the power of curing them, are totally distinct, and ought to be fully possessed by every man who aspires to the character of a surgeon; and we conceive that, by supporting this opinion, and endeavouring to restore to surgery this extensive and important branch of practice, the profession cannot sufficiently appreciate the labours of the late Mr.

SAUNDERS especially, of Mr. TRAVERS and Mr. LAWRENCE, of Dr. VETCH and Mr. GUTHRIE.

The Preface terminates by an announcement, that the Lectures on the Anatomy and remaining Diseases of the Eye will follow the completion of a work, in which our author has been for some time engaged, on the Diseases of the Urethra.

The first subject treated of in the work before us is the *Inversion of the Eyelids*, and the various operations that have successively been employed for its cure. We pass over the description and history of this disease, which are accurately and vividly given, but are too long for insertion. We find our author in part giving to Mr. CRAMPTON the merit of having first pointed out the principal causes of this disease, and as having also indicated the proper method of cure; but, as Mr. Guthrie disagrees with that gentleman in some of the minuter points of his explanation, we shall give our author's summing-up in his own words:

"1. That the derangement of the eyelashes, constituting trichiasis, is frequently the consequence of disease; but seldom or never arises from unnatural formation, or from an accidental or too luxuriant growth of the cilia.

"2. The relaxation of the integuments, or a partial paralysis of the levator palpebræ, is a natural and otherwise healthy state of the eye and eyelids, are not primarily concerned in the formation of entropium, and never alone give rise to it; although, if other derangement take place, they may (by removing some power of resistance to it) assist in its more complete formation.

"3. That, in a general or complete inversion of the edge of the tarsus of the upper lid, the swelling of the external parts, and the spasmodic action of the orbicularis palpebrarum, first tend to the formation of the disease, and which is completely established by the contraction of the angles of the lids, and the accidental inversion of such hairs as become stiff and matted by the vitiated discharge from the meibomian glands and conjunctiva.

"4. That the change in the curvature of the lid takes place principally from the contraction of the angles, whilst under the influence of the orbicularis, and not from the contraction of the conjunctiva, corresponding to the superior broad ligament which supports the tarsus, and maintains the shape of the upper eyelid.

"These conclusions do not, of course, apply to such partial cases of trichiasis as result from the formation of a cicatrix, tumor, or other direct and obvious cause, to which I have already alluded." (p. 15.)

The methods of remedying this distressing complaint are next described; and there are few diseases, observes our author, in which so little alteration in the method of operating has taken place since the time of HIPPOCRATES: but this remark does not appear to us to be borne out by the facts; for, although the *principle* has necessarily been the same in every age, the mode now in use is surely very different, and far superior to that practised by the Father of Medicine. We must be excused from enumerating the various plans that have been proposed to effect the same object. The reader will find the result of much research in this part of our author's work; but we must pass over this portion of the subject rather hastily, in order that we may detail the operation as recommended by Mr. Guthrie. We may, however, observe that the German writers are extremely divided as to the propriety of performing that operation, (namely, a removal of part of a fold of the skin of the eyelids,) by which a radical cure is effected, or the more simple one of occasionally removing the offending hairs. Weller and Beer support this opinion, whilst Dr. Jaeger proposes the removal of the edge of the eyelid, including the cilia; but which, as our author remarks, differs in no respect from the operation recommended by Mr. Saunders many years prior to Dr. Jaeger's publication. Our author says (page 25,) that he has seen three patients on whom this operation had been performed : in all, the deformity was considerable, and the relief partial. Passing by an improved mode of operating introduced by Mr. Crampton, and some remarks upon the method proposed by Quadri, we come to the description of the author's operation, and which he recommends as equal to the cure of the worst cases.

"The operation I recommend, as equal to the cure of the worst cases, is to be performed in the following manner:—The head being properly supported, the eyelids are to be gently separated; the patient is to be desired to refrain from making any effort whatsoever, and the surgeon is to wait until he sees that the lids are perfectly quiescent. A small narrow knife, or one blade of a blunt-pointed scissors, is then to be introduced close to the external angle, and a perpendicular incision made, of from a quarter to half an inch in extent, or of a sufficient length to render the eyelid quite free: the quiescent state of the lids, and especially of the orbicularis muscle, enabling the surgeon to cut closer to the angle than he otherwise could do, and thus to divide the ligament, or at least the extremity of the cartilage. Another incision is to be made, in a similar way, at the inner angle; but this should not include the punctum lachrymale, as I have never found it necessary to do so; and, although the tears may continue to pass through the lateral canal into the sac when the punctum has been included in the incision, they do not do so with equal freedom, and there is some observable deformity. The length to which the perpendicular incisions at both angles ought to extend, must now be decided upon by the appearance of the part; they must be continued, if necessary, by repeated touches with the scissors, until that part of the eyelid containing the tarsal cartilage is perfectly free, and is evidently not acted upon by the fibres of the orbicularis muscle which lie upon it. This frequently causes the incisions, and especially the internal one, to be longer than is usually supposed to be necessary. The part included in the incisions is now to be completely everted, and retained by the fore finger of the operator's left hand against the brow of the patient; when, if any lateral attachment be observed, acting upon, and drawing or confining the lid, it is to be divided, which is in fact still elongating the incision. On letting the eyelid fall on the eye, the edge of the tarsus and the hairs will frequently appear in the natural situation, in consequence of the relaxation of the angles which bound them down; but, if the tarsal cartilage has become altered in its curvature, this will be immediately perceived: it will turn inwards at its ciliary edge, and be completely bent at its extremities, more especially at the inner one, where it is more powerfully acted upon by the musculus ciliaris. On desiring the patient to raise the lid, he readily attempts it, but the action of the levator, in such cases of vicious curvature, causes the cartilage partly to resume its situation; and, on examination, the curve will be observed to be so permanently vicious for about an eighth of an inch at each extremity, and especially at the inner, that it cannot be induced to resume its actual situation. When this is the case, the cartilage is to be divided, exactly at the place where it is bent, in its length, and in a direction at a right angle with the perpendicular incision. The portion thus slit is only connected with the common integuments of the eyelid; and, although this incision scarcely exceeds one, and never two-eighths of an inch, at both extremities, and in general is only necessary at the inner, it enables the surgeon to remove the altered curvature of the part.

"The operation being thus far accomplished, a fold of skin is to be cut away from that part of the eyelid included between the incisions; three or four ligatures are then to be introduced, and the divided parts from which the fold has been removed, are to be neatly brought together by the ligatures, each of which ought to be twisted, and then fastened to the forehead by several short slips of sticking-plaster, the ends being turned over the plasters near the hair, and retained in that situation, to prevent their slipping. In raising the fold of skin, care should be taken to do it regularly with the fingers; it is also essential to the success of this part of the operation, that it be done as close as possible to the margin of the eyelid. It may then be grasped by the forceps of Beer,* which have transverse pieces, slightly curved for the purpose at their extremities, and close with a spring. The piece thus included, which need not be large, may be cut away at one or more strokes of a large pair of curved or straight scissors. The ligatures should be inserted first at each angle; and, when the vicious curvature is considerable, I not only pass it through the skin, but take care that the internal one shall include, at its lower part, the outer edge of the margin of the eyelid, which, from its firmness, retains that ligature much longer than those which are passed through the skin only, and tends to prevent the possibility of a relapse. The ligatures thus placed are to be equally drawn up on the forehead, until the eyelid is com-

* "To the forceps used by Beer and Langenbeck, Messrs. Everill and Mason have adapted the spring used by Gibson for his artificial pupil forceps, which causes them to secure the raised fold without pressure by the operator.

1

pletely everted, when they are to be fastened as directed. In order to prevent any attempt at union but by granulation or a filling up of the incision, the edges are to be slightly touched with the sulphas cupri. The eye and eyelids are now to be carefully cleansed; a piece of lint, spread with the unguentum cetacei, is to be placed upon them; a small compress is to be put under the edge of the eye bone and orbit; a retaining bandage covers the whole, and completes the different steps of the operation. When the disease is not considered of an inveterate nature, the ligatures may be introduced through the fold of skin, without cutting any portion away. They give little additional pain, and are infinitely more effectual than any other suspensorium with which I am acquainted, and they ought to be assisted by strips of adhesive plaster placed between them. The operation, accomplished with all the care I have described, will still fail, if equal attention be not daily paid to the subsequent dressing, on which, indeed, more depends than on the operation itself; so much, indeed, that I am disposed to consider inattention to it the most certain cause of failure. In more than seventy operations that I have performed, I have never known inflammation or other bad consequences ensue. The patient is to be kept perfectly quiet until the next morning, when the bandage and lint are to be removed, the eye carefully fomented and cleansed with warm water, and the dressings replaced. On the second day, great attention must be paid that the ligatures keep the lid sufficiently raised; and, if any union has taken place by adhesion at the angles of the incisions, it must be broken through with the probe. On the third day, the plasters attaching the ligatures to the forehead will in general require to be exchanged. The ligatures themselves must be supported by straps of plaster placed vertically between them; the edges of the incisions should again be touched with the sulphas cupri, or separated by the probe. The great art of the cure now consists in causing the incisions to be filled up by granulations only, so that the eyelid may be lengthened as much as possible, and which can only be effected by a continuance of the means indicated. In a few days more, and especially by the continued elevation of the lid, the ligatures cut their way out, during which period the eyelid is gradually lowered; and, by the time the incisions have filled up, it will have resumed its natural situation, and the cure will be completed. There will, in general, be an indentation in the ciliary margin of the tarsus, where the incisions were made; but this is sometimes scarcely perceptible, and never detrimental. If the patient should have kept the lids closed at the commencement of the operation by the action of the orbicularis, the first incision may not have been made as close as might be desired to the angle of the lids; and in this case a hair or two at that part may not afterwards obtain quite its or their original direction, and cause some inconvenience; but this does not often happen, as these hairs are usually carried clear of the eye." (p. 31—35.)

The operation on the under eyelid is analogous to that on the upper, but less severe, as the parts are more simple: we have not room to describe it. These operations are illustrated by six cases.

A few pages are next devoted to a consideration of the *Relaxation of the Upper Lid;* the causes of which complaint being various, the remedy will, of course, depend upon the proper treatment of the disease of which this is generally a symptom. We may dismiss this subject by observing that, when this affection arises from palsy, the cure is very uncertain, and the prognosis, of course, unfavourable.

We now proceed to examine the next section, on the *Eversion of the Eyelids,* and which, for the sake of perspicuity, our author has divided into four distinct species as follow:

"1. As depending on chronic inflammation, accompanied by contraction of the skin and of the integuments of the lid; but without any marked cicatrix.

"2. As depending on acute inflammation, or immediately following it, with relaxation or swelling of the conjunctiva.

"3. As depending on the contraction occasioned by a cicatrix, on the healing of a wound, on or in the immediate vicinity of the eyelid.

"4. On paralysis."

This disease is a minor inconvenience compared to the inversion of the eyelids, and is met with in every degree, from the mere dropping of the tears to that stage in which the cornea is opaque from the effect of chronic inflammation, and the conjunctiva is thickened and dried: that portion of it forming the fold between the eyeball and lower lid may be so swollen as to form a substance almost horny between them; at another time the whole conjunctiva will protrude, so as to hide the ball of the eye. We pass over an account of the various modes of remedying this deformity, practised from the time of Hippocrates to the publication of BORDENAVE's paper in the Memoirs of the French Academy. We regret, also, that we can only refer to the work before us for an account of the methods of treating the first and second species of this complaint, as described by our author. With respect to the third species,—viz. that depending on the contraction occasioned by a cicatrix, or the healing of a wound in the immediate vicinity of the eyelid,—we find the following important remarks:

"In this species, the appearances and actual situation of parts differ materially from the other two, and are influenced by different circumstances. It is well known that the first step towards the cure of an ulcer or wound, is the drawing in of the surrounding parts, in a manner commensurate with the growth of the granulations which are to fill up the vacuity that has been made by the progress of the ulcer; and, by the time cicatrization has taken place, the cicatrix bears no proportion to the size of the original wound. But this process of contraction does not stop here; it continues after the ulcer has healed, especially in loose parts; and, in burns of the neck, it is capable of drawing down and fastening the chin to the breast. This diminution in the quantity of new-formed parts continues until there is little left to mark where the solution of continuity existed: but this little does not resemble the original structure; it is firmer, harder, more stringy, and somewhat resembling the nature of ligament. The cure has been effected at the expense of the surrounding parts, which, if loose, as in the neck or in the eyelid, may be drawn in to a very considerable extent. In consequence of a knowledge of this fact, our attention, in cases of burns in the neck, should be invariably directed to the position of the head, which must be kept backwards until long after the wound has healed, in order to counteract, as much and as mechanically as possible, this tendency to contraction, and to force nature to form more new skin or a larger cicatrix, which she is always very unwilling to do, and is often indeed incapable of doing. The same thing takes place on the eyelid; and, if an ulcer be allowed to heal quickly, eversion, in a greater or less degree, is an invariable consequence. In the first and second species, the operation of this cause is slight, and on the edge of the eyelid or cartilage; in the third it is greater, and on the whole external surface of the cartilage, which is turned downwards, whilst the eyeball is more exposed: the whole of the conjunctiva of the under lid is distinctly seen, and forms something resembling an inclined plane from its angle of reflection over the eyeball, outwards and downwards. The deformity is more striking. In the second and third species, the conjunctiva always forms more or less of a sulcus, or hollow, with the eyeball at the angle of reflection: in the third, this part is fully exposed, the conjunctiva is drawn downwards; the tears always flow over the cheek; the curvature of the tarsus is altered; the ligaments are more stretched, and the elongation of the eyelid becomes more apparent; the conjunctiva is sometimes fungous or granulated, and occasionally but little affected or altered, but in many instances it assumes a lippitudinous appearance; the cicatrix is hard, and, for the most part, firmly attached to the cheek-bone.

"It was for the cure of this species of the complaint that Bordenave, whose memoir I have already noticed, recommended the double operation of the removal of the cicatrix and of the fold of the conjunctiva; and, although he did not succeed in effecting a perfect cure, still he so much relieved his patients as to render his method deserving of the greatest attention: it will, however, be observed, it was only returning in some measure to the practice of the ancients. That it was not sufficient, is attested by the subsequent observations of Scarpa and Beer, who declare this species, in severe cases, to be incurable." (p. 71—73.)

To effect a cure of this species of eversion, continues our author, it is necessary first to remove the cicatrix of the old wound, with the strong membranous threads which attach it to the bone beneath, and then to give such permanent support to the eyelid as will enable it to resist the contractile force of the granulations from the cicatrization of the wound below.

"The operation for the third species consists, first, in the removal of the adhesions which the cicatrix has formed to the cheek-bone, with the whole or such part of the cicatrix as can be with propriety taken away; secondly, in the cutting out of an angular portion of the eyelid, near the external canthus; thirdly, in the removal of the diseased fold of the conjunctiva, if there be any,) by a horizontal stroke of the scissors, assisted by the forceps; fourthly, in the application of two sutures, to bring the divided edges of the eyelid together,—one close to the margin of the lid, the other near the point of the angle below: these are to be drawn just so tight as to bring the parts in close apposition, when they are to be cut off close to the knots, and supported by slips of sticking-plaster; fifthly, in the dressing of the wound made by the removal of the adhesions to the bottom with very fine lint, and applying a bandage over the whole.

"The subsequent dressings require attention. The object intended by the sutures will almost always be effected by the third day, when the edges of the angular wound will be seen to adhere, and the sutures are really of no further service, whilst a retention of them in the part would lead to ulceration. They are therefore to be cut and the threads removed, which will allow the openings made for their reception to close. Strips of adhesive-plaster should be applied in their place, and a small compress, to give additional support to the part. If the edges of the lid should not have perfectly united, a small notch will remain for a short time, but which will be gradually filled up. If the lower part or apex of the angular piece should not have been consolidated by adhesive inflammation, it will fill up by granulation, which may be assisted after a time, or any fungus repressed, by gentle touches of the argentum nitratum.

"The wound made by the removal of the cicatrix, or of the points of attachment to the cheek-bone, is to be kept open after the first dressing, by an ointment composed of equal parts of the ung. lyttæ and of the ung. resinæ flavæ, and it is not to be allowed to close until the granulations have reached the level of the surrounding parts. During the process of cicatrization, the eyelid must be carefully supported by strips of sticking-plaster.

"Slight inflammations accompanied by pain may, although in general they do not, take place: in such cases, the compress and bandage placed over the eye to complete the dressing ought to be kept wet with cold water, and a cathartic should always be administered on the morning subsequent to the operation. Poultices should be avoided, particularly in the third species, as they tend to promote suppuration and prevent adhesion." (p. 77—78.)

Of the fourth species we need not say much, as the treatment must be directed to the cure of the general disease; and, in truth, but little can be expected in such cases. We quote the following case in illustration of the operation proposed for the cure of the third species:

"John Smith, 22d Foot, admitted, labouring under complete eversion of the lower lid of the left eye, occasioned by an extensive wound he received fourteen years ago, by the wheel of a cart passing over the side of his head, and fracturing the frontal bone and the external margin of the orbit. The edge of the everted tarsus adheres to the malar bone by a firm cicatrix, and the muscles of the cheek exercise great power in drawing it downwards whenever the lower jaw moves. He has been in this state fourteen years, and suffers much inconvenience from it.

"23d. The operation for ectropium performed by making a free horizontal incision beneath the whole ciliary margin of the lid, and thus detaching it from its first adhesion to the malar bone; after which a triangular piece was cut out of the centre of the tarsus, and the lips of the wound brought together by two sutures. The lid was then retained in its natural position by adhesive straps and a bandage.

"25th. The dressings removed this morning; the granulations of the horizontal wound healthy; has experienced no pain since the operation.

"27th. The wound of the tarsus perfectly healed; the sutures removed; granulations spring up rather too abundantly from the horizontal incision beneath the orbit, and were therefore touched with the sulphate of copper.

"March 6th. The wound nearly healed.

"10th. Discharged; the lid perfectly restored to its natural position." (p. 81.)

We shall pass over a few pages devoted to the consideration of *wounds of the eyelids*, and of various small tumors to which they are liable; all of them distinguished by a particular name, mostly of a very formidable length, and of Grecian contour, the frequent occurrence of which we cannot but lament, as tending sadly to obscure a subject sufficiently difficult in itself: indeed, if the minute and almost imperceptible shades of difference in diseases of other parts of the body were equally disfigured or disguised by distinct names, the whole period of a medical education would scarcely be sufficient to learn the mere language of the science. What shall we say to such words as the following—*ptilosis*, *pachytes*, *pacheablepharosis*, and *pacheableophora?* the simple meaning of all which is, that the cilia have fallen out, and the edge of the eyelid is thickened.

On the *Encanthis*, or enlargement of the caruncula lacrymalis, we need not dwell long: the disease, according to the testimony of Mr. Travers and our author, is not of very frequent occurrence in this country, and is for the most part the consequence of direct injury to the part, and of course affords the usual symptoms of inflammation, ending generally in the formation of a small abscess in the part, and from which (though rarely in this country, at least,) a fungus sometimes springs up. In the treatment of this affection, it is necessary to recollect that the inflammation often proceeds from the presence of a foreign body,—such as a loose eyelash sticking in the upper punctum, and irritating the caruncle. This must, of course, be removed; and the application of leeches to the part is very properly recommended, together with the fomentations of warm water. Our author condemns, and in our opinion with great reason, the use of cold applications, as advocated by the continental writers. If the fungous growth above mentioned should follow the formation of the abscess, the infusum sabinæ, or the metallic escharotics, are recommended for its removal. When the fringed-like excrescences, or fungous growths, are the consequences of inflammation which has not proceeded to suppuration, they should be cut off with the scissors, and the roots touched, in a day or two after, with the sulphate of copper. When the encanthis is not inflammatory, it may be removed by the knife or scissors: the only precaution necessary to be observed, is that the caruncle itself should be left entire, or, at most, as little of its substance removed as is possible.

We must be contented merely with saying that the *Pterygium* is next described, in both its varieties, and the mode of extirpating it related.

The next section is devoted to the consideration of the means of removing *Extraneous Substances from the Eye*, and of *Injuries of the Eyeball*; and the observations which Mr. Guthrie makes on these subjects are highly judicious and instructive; but we can do little more than recommend them to the attention of the student, and proceed to more important matter.

*Of Tumors which are formed within the Orbit, and on Protrusion of the Eyeball.*—These tumors, says our author, are almost always encysted, and are distinguished from the effects of inflammation and abscess within the orbit, by the absence of any violent symptoms; and a case is given from RICHERAND to show the usual progress of such tumors. The best method of treating them, if deeply seated, appearing to be that of making a small opening into the tumor, and letting out its contents. Suppuration is induced by cutting off a portion of the sac, and introducing a piece of lint at the bottom of it. If the tumor be composed of a fatty substance, after the division of the conjunctiva over it, it may be laid hold of with a double hook, and dissected out, and which will be readily effected under such circumstance.

We lament extremely that the subsequent part of this section, upon the removal of tumors deeply seated within the orbit, and on the protrusion of the eye-ball, are too long for insertion, and do not conveniently admit of being abridged.

The subject of *Cataract* is next considered: it occupies upwards of two hundred pages of closely-printed letter-press, presenting a clear and luminous account of the opinions and practice both of the ancients and moderns, and entering minutely into the discussion of some of the nicer points which have produced much difference of opinion among the writers of our own country, as well as on the continent. It is obviously impossible for us to give any satisfactory analysis of this portion of our author's labours: we have, however, ventured to make a few extracts, *en passant*, from those parts which appear to us to possess most novelty, or to be of peculiar interest; and the effect of which can only be that of exciting the curiosity of our readers to examine the volume itself. We find nothing to notice particularly in the symptoms of cataract. On the subject of the diagnosis between that disease and incipient and confirmed amaurosis, we find the following remarks:

"The incipient amaurosis is occasionally accompanied by some of the symptoms attributed to muscæ volitantes; but one or more of the spots will in general be found to be fixed, of a darker colour, and permanent, whilst a general indistinctness of vision takes place and increases. This is, in other persons, only discoverable at first after a steady application of the eye to a particular object, when the sight is suddenly found to fail altogether, but returning again after resting the eye; or the patient describes himself to have suffered the loss of half of his sight, so as to be able to see only the half of an object; both of which states are, for the most part, accompanied by more or less pain in the forehead, temple, or eye. These changes are sometimes periodical. The sight is not improved by the use of spectacles, or by the application of the belladonna; seldom by viewing an object sideways, or to the right or left of the direct axis of vision, which it is in the early stages of cataract. Flashes of light, or white and brilliant circles, or luminous spots, equally indicate amaurosis; and the change in the colour of the flame of a candle from white to yellow, or red, or green, is also demonstrative of that state; in which the patient occasionally sees, when the eye is closed during the night, luminous circles of different colours. The halo which is observed to surround the candle in cataract, of a white clouded colour, is in amaurosis of a bright yellow, or red, or green, the colours being more confused as the object is more distant. The cornea, in the incipient amaurosis, is scarcely altered from its natural state; but, at a later period of the disease, it evidently loses its perfect transparency and clearness, and becomes rather turbid or dull in appearance. The iris is at first sluggish in its movements, or the pupil has even begun to be dilated; at which period little or no change can be observed either in the lens or vitreous humour: but, as the disease advances, the pupil becomes more dilated, the edge of the iris loses its regularity, the pupil is no longer round, but irregular, or angular and fixed. In that form of amaurosis in which the actions of the iris and the motions of the pupil remain unaffected, no opacity is perceived behind the pupil which can be mistaken for cataract. The cloudiness which occurs in amaurosis is always distant from the iris, and is for the most part accompanied by a general change in the appearance of the lens and vitreous humour, which no longer seem to be of a perfectly jet-black colour, but are changed so as to allow the bottom of the eye to be seen through them, of a pale greenish or horn colour, giving to the whole somewhat of the same hue. The cloudiness observed behind the pupil is deep-seated, and, on a careful examination in every direction, particularly sideways, may be observed to have a concave appearance, dependent on the form of the posterior part of the eye. If a particularly opaque spot can be observed, when the pupil is completely dilated, it will appear of a greenish or reddish-green colour, but not grey as in cataract; and, by changing the position of the eye, it will be found to change its place also, being caused by the refraction of light, and not by any permanent opacity. The eye will frequently be softer than natural, in which case the motions of the iris may be tremulous; the vitreous humour will then be thinner than usual, or even be dissolved." (p. 212, 213.)

In distinguishing cataract from that alteration of the vitreous humour, accompanied with an alteration in the structure of the hyaloid membrane, of the retina, and tunica choroidea, which is termed *glaucoma*, the following differences are usually observable. In this latter affection, the lens is not implicated, at least for a very considerable time.

"When the disease is fully formed, so as to be confounded with cataract, or to render a diagnosis necessary in order to prevent a useless and dangerous operation, the symptoms are as follow:—The eye has a general unhealthy appearance, arising from a turbid state of the cornea, which has lost its brilliancy, although in no one part has it become opaque. The sclerotica does not preserve its natural appearance, being either more of a bluish or yellowish colour, whilst several tortuous dark-red vessels may be observed, especially on the under and upper part of the eye, which do not run on to the cornea, but penetrate the sclerotica at a distance not exceeding the eighth of an inch from it, and sometimes less, giving rise, when they are numerous, to the appearance of a white ring or circle situated between them and the cornea. They are varicose vessels, coming from within the eye, and intimately connected with varicosity of the vessels of the choroid coat; and, as that state of disease increases, they assume a darker red or blue colour, become larger and more tortuous, often communicating with each other where they penetrate the cornea, forming also a sort of vascular ring, exterior to the bluish-white one already noticed. If the eye is examined by the touch, it will be found rather firmer or harder than natural.

"The iris, if only one eye is affected, differs a little from its natural colour: if formerly blue, it has now become grey; a black iris changes to a dirty brown; but this alteration is of no consequence compared with the state of the pupil, which is dilated, unless inflammation of the iris, which is not an essential characteristic of the disease, has been superadded to it. The dilatation of the pupil is always accompanied by a marked irregularity of its edge, sometimes rendering it angular; whilst it is always perfectly fixed or immoveable, and occasionally drawn to one side, sometimes to both, rendering the pupil oval. The patient cannot distinguish light from darkness. (p. 214, 215.)

Respecting the causes of cataract, our author gives us only the usual explanation; and he then presents us with a classification of cataracts, which he has adopted in order to simplify the consideration of their diagnosis: he accordingly divides them into two classes, the true and the false. The first class has three genera: the first is the lenticular cataract; the second, the capsular cataract; and the third, the capsulo-lenticular. The second class, or the false cataracts, contain all those which are combined or connected with derangement of the iris or adjacent parts, as a consequence resulting from inflammation, (p. 227:) each of these combinations is described, but we have not room for the discussion; nor can we promise to our readers any advantage from the insertion of any farther extracts that we might be enabled to make from the remaining portion of this work.

The chapter on the *Formation of Artificial Pupil* is alone of a length and intricacy to defy compression within any moderate compass, so that our readers should derive either pleasure or profit from our labours. We can only add, that those to whom this subject is interesting, including, we believe and hope, the whole rising generation of surgeons, will find ample details respecting all the disputed points,—all the advantages, real or supposed, of the plans that have been proposed or modified by so many different writers; and we moreover think that they are discussed with a spirit of impartiality and candour, very much to Mr. Guthrie's credit. That gentleman will, no doubt, excuse our hasty and meagre notice of his book, because the impossibility of our going through it seriatim, without extending this article through three or more Numbers, will be apparent to him; and we therefore take our leave of his work, by again recommending it to the perusal of the profession.

The plates which accompany this volume are in general well executed; but there is one error which it is incumbent upon us to advert to, because it is of a nature to raise a doubt as to the authenticity of the engraving, and should be corrected or explained in a subsequent edition. It is in the 6th and 7th figures of Plate III.; the first representing a strongly-marked case of glaucoma, the lens being scarcely affected; the second (fig. 7,) is said to be *the other eye of the same person,* in which the lens is of a mahogany colour, &c.—whereas, unfortunately, *both the above are left eyes.*

---

Art. II.—Drs. Campbell, Mackintosh, and Douglas, *on Puerperal Fever,* (concluded from page 226.)

Dr. Campbell seems not to be sufficiently aware of the great distinction between infection and contagion. He asserts that puerperal fever is not *infectious,* and we believe him. No more is the plague *infectious,* but it is highly *contagious;* and so is puerperal fever. This is not the place for renewing the long-agitated question of infection and contagion as two distinct actions. We have done this, and, we thought, to the satisfaction of all unprejudiced readers, on a former and memorable occasion, in a work written professedly on the subject. We must, therefore, at present limit ourselves to simply re-asserting, that puerperal fever (not the plain peritonitis of either sex,) is contagious: that is, it may be contracted by the same means that the plague is. Characters of *infection* it has none. We have never been able to trace any in it, and we have seen some few cases of the disease. Well may Dr. Campbell smile at those who talk of the infectious nature of the disease in question, if by that word it is meant to say that the disease is communicable by atmospheric transmission. Such a notion is, in fact, ridiculous. But it is as ridiculous to state, in the way of a triumphant answer to those who contend for the *contagion* of the disorder, that cases occur, for the appearance of which they could not account, no communication having been traced between the patients. Does the admission of a disease being contagious, then, preclude the possibility of its appearing likewise sporadically? And, if it has appeared sporadically in A, why can it not also make its appearance in B placed under similar circumstances, though beyond all possibility of communication with the former? In fact, all contagious diseases *must* have begun sporadically, and may do so, either in one case only, or in a thousand, else "*unde origo mali?*" Is it too great a stretch of the imagination after this to believe (what indeed is the fact,) that some few such diseases,—take, for example, the plague, or some species of typhus,—may be farther propagated by bringing an individual labouring under them in *immediate* or *mediate* communication with another in a state of health? Precisely such a process obtains in the puerperal fever,—not the simple peritonitis of either sex, we repeat. Let us take our illustrations from Dr. Campbell's own cases. Mrs. Nielson (Case 1,) is attacked with fever on the third day, is attended by Dr. Campbell, and she dies. This may have been a sporadic case, just as much as any other which may have happened, for ought we know, to other practitioners on the same day, but in different parts of the town: there is nothing unnatural in this. Mrs. Macdonald (Case 2,) is also taken ill with fever a few hours after delivery, the very day on which the dissection of Mrs. Nielson had taken place. Dr. Murphy, who attended Mrs. Macdonald during her labour, had also visited Mrs. Nielson during the fatal progress of the fever; and Dr. Campbell, who had been narrowly watching Mrs. Nielson during her complaint, also visited Mrs. Macdonald on being taken in labour. This did not, however, take place till three days after, and several hours after blood had been taken from the arm to eighteen ounces. The two cases went on simultaneously; that is, the progress of the fever in the one was contemporary with the progress of the labour in the other; and both were alternately attended by the same practitioners. We do not wish to be hasty in drawing conclusions from this singular approximation; but what is there in it that should prevent us from suspecting that the one case (Mrs. Macdonald's) might possibly have been the result of *conveyed contagion?* At all events, the contemplation of these cases, (the two very first on Dr. Campbell's list,) does not authorise that gentleman in asserting that nothing had happened in his practice to warrant the suspicion of infection,—if by that word be meant contagion. We might extend the same observations to several more cases, particularly to those delivered in October 1821; but *verbum sat.*

We have no room for any further observations on this subject; but we agree with our author that the fever in question may prevail epidemically, owing to some particular condition of the atmosphere, affecting equally a great number of individuals, at or about the same time, without any communication, infection, or contagion.

Before we dismiss the subject of contagion in puerperal fever, we will quote an anecdote which has been related to us by one of the parties. A general practitioner, in one of the neighbouring villages of London, applied, twenty years ago, in great agony of mind, to one of the leading—indeed, most eminent—accoucheurs of the metropolis, not more remarkable for his skill and long-tried experience, than for his candour, friendly disposition, and humanity, for advice under the following distressing circumstances. He had lately attended several cases of labour, and had successively lost six patients of puerperal fever. The poor man dreaded the very idea of another labour, and was preparing to quit the place in which he had long practised. What, under such distressing circumstances, was to be done? "I tell you what, Mr. ——, (says the accoucheur;) go home, and make a bonfire of every rag you possess, and get you new clothes." Some time after this, the accoucheur was surprised to hear himself addressed in the style of acquaintanceship by a person whom he thought he did not know. "Sir, (said the stranger,) you recollect, without doubt, giving a singular advice (meant to imply much,) to a general practitioner on the subject of puerperal fever?"—"To be sure I do, very well."—"That practitioner *I* am. I did then as you suggested, and never had a case of puerperal fever afterwards."

There is a practical lesson in this anecdote, which *we* shall never forget; however hardily Dr. Campbell and his colleagues may talk of their "having used *scarcely* any precautions to prevent the infection from spreading" in the circle of their practice.

*Pathology of Puerperal Fever.*—We cannot enter into the learned dissertation of our author respecting the seat of this disease. It appears evident to us, from what we have read, that the uterus is the principal seat of the disease assumed by Dr. Campbell, though he does not pretend to decide whether the disease first begins in the peritoneal coat, or in the substance of that viscus. He is inclined to suppose that it sometimes commences in the one, and sometimes in the other. From the number of dissections we have seen of patients who died of this complaint, at home as well as abroad, we are inclined to think that the portion of the peritoneum which covers the posterior part of the uterus is most commonly affected at first, and that the inflammatory action progressively spreads to either of the broad ligaments, and the uterine appendages embraced by them, as well as to the intestines lying the nearest to the inflamed portion of the uterine peritoneum. This opinion we published some years ago, and the experience of subsequent years has tended strongly to confirm it.

Come we now on more debatable ground; and we feel the less hesitation in grappling with such an antagonist as Dr. Campbell, as, from all we read in his book, we have formed a favourable judgment of his medical and moral qualifications, a little misguided here and there,—a *tant soit peu opiniâtre* in his own way of thinking,—but zealous, candid, and warmly attached to his profession.

Before we discuss the nature of puerperal fever with Dr. Campbell, it will be well to recal to our readers the opinions of an author, to whose Essay on Puerperal Fever we briefly alluded in a former Number: we mean Dr. Douglas, of Dublin; and in so doing we present them with no mean authority, as they must be aware of, if they have attended to his former writings.

Dr. Douglas was addressed, by the General Board of Health in Ireland, on the subject of puerperal fever, early in the year 1820. The information sought after was indicated in a number of queries put by the Board to the Doctor; which queries, embracing the principal objects of the investigation on puerperal fever, together with the Doctor's answers, have been published in the third volume of the "Dublin Hospital Reports," now before us.

The opportunities which Dr. Douglas has had of studying this disease have been ample. He resided as assistant in the Dublin Lying-in Hospital for three years, 1809, 10, 11; during the winter of one of which (1810-11) the fever visited the hospital in a malignant form; since which time his experience has increased with his practice. He states that he has never seen a truly epidemical form of the disease any where, except in the Lying-in Hospital; yet, during his residence in Dublin, few years have elapsed without his prescribing for some cases of that species of the disease usually termed puerperal peritonitis. Dr. Douglas very justly observes that, in the greater number of instances, this sporadic puerperal fever may, if judiciously treated, be subdued in the course of a few days. The fatal cases of it, whether in hospital or private practice, he enters at scarcely one in six; whereas, in the truly epidemic fever, hardly one-half of those absolutely attacked recover. This species of the disease he does not consider as infectious.

It is satisfactory to find that these opinions coincide with those of other practitioners, who, from their situation, must have an equally extended field of experience with Dr. Douglas in simple or sporadic *peritonitis post partum*. In the Report of the Practice of Midwifery for 1818, at the Westminster General Dispensary, to which we have before alluded, there are observations precisely similar to those of Dr. Douglas, respecting the fever in question. It is there stated to be a very prevalent complaint among lying-in women; to consist in a general or partial inflammation of the peritoneum; to be common to both sexes, but more particularly to affect the woman in child-bed, as well as her who has miscarried: to be readily subdued by bleeding, which is there called "the heroic remedy for this disease;" and, lastly, not to be contagious,—meaning the simple peritonitis. Indeed, Dr. Campbell himself agrees with both Dr. Douglas and the author of the Report, as far as the consideration, nature, and treatment, of simple peritoneal inflammation after labour extends. But things are far different when this inflammatory fever, either by complication or aggravated modification, assumes a more formidable character. Here Dr. Douglas's extensive experience is at variance with Dr. Campbell's sweeping assertions; and we are told by the former that it is then malignant and contagious.

"It is customary to apply the term puerperal fever to diseases very considerably, although not entirely, different; different both with respect to their exciting cause and as to the type of their pyrexia, although always similar in their great leading characteristic,—abdominal inflammation and pain. I have for many years been of opinion, that there is not a greater difference in the type of that pyrexia which accompanies a case of any purely inflammatory disease, as phrenitis, pleuritis, &c.—and from such down, through every grade of fever, to the plague itself, —than there is between the type of the pyrexia attending a casual* case

* "By the term casual case of puerperal fever, I mean such as occasionally occurs, both in hospital and private practice, when the disease is not epidemic, and which is excited by accidental causes, either during labour or subsequently to delivery. In these cases, I presume the local inflammation more frequently to be primary, and the pyrexia to be consequential; whereas, in the epidemical, I consider the pyrexia to be primary, and the local affection to be consequential."

of puerperal fever, and of that attendant on the true puerperal epidemic; and I feel myself justified in attributing the discrepancies of opinion every where existing with regard to their nature, and the various, and even opposite, modes of treatment recommended by different authors for the cure of puerperal fever, to deficiency of nosological distinction." (*Dublin Hospital Reports*, p. 143, 144.)

Having distinctly stated what *real* puerperal fever is, in all which we coincide, let us hear what Dr. Douglas says farther about contagion.

"When puerperal fever is epidemic, I consider it to be really contagious, but, for the most part, only to lying-in women: I do, however, believe that a woman, either pregnant or whilst nursing, or even a very delicate female, for several months after lying-in, although not nursing or pregnant, might be susceptible of this disease. I likewise think that any woman, whether married or single, at particular periods, might be liable to an attack of it, if much exposed to the influence of an hospital epidemic. Cases, occurring under these different circumstances, have happened within my own knowledge. I am rather apprehensive that such contagion may be conveyed by persons much engaged in hospital duty, at a time when its atmosphere* is heavily loaded with this peculiar effluvium. I have been informed of the circumstance of a pupil in midwifery having remarked, at the period of an epidemic, that several patients successively, upon whom he waited during labour, were seized with this disease, and died. Now it is more than possible that those women might have been infected from the contaminated atmosphere, or by some other widely operative cause; but the young gentleman became so apprehensive that he himself had been the medium of conveying the infection, that he resolved not to attend upon any other patient during the prevalence of the epidemic." (*Idem*, p. 144, 145.)

We are now prepared to discuss the question respecting the pathology of the disease. Dr. Campbell, having embraced an exclusive opinion with regard to the nature of childbed fever, enumerates one by one, and analyses, the opinions of those writers from whom he differs; and the result of all his observations clearly is, that what he has considered and treated under the appellation of puerperal fever does not, in the slightest degree, vary from common peritoneal inflammation. He contends that the symptoms announcing the invasion of the disease,—those which mark its progress,—and the appearances after death, collectively as well as separately, corroborate the justice of such an opinion.

He will not admit that the disease he has in view can by any modification become different, or present distinct characters, from those which attend it in its simple form, as seen by him; and, although he somewhat reluctantly concedes even to his

* "I have readily perceived, by the sense of smell, the effluvia of puerperal fever, when epidemic, on entering a ward of the hospital."

simple peritonitis the liability to become putrid in its progress, (p. 180,) he contends that this is no more than what may be remarked in every other fatal case of inflammation. "To assert that it is so at the commencement, is a dereliction of truth."

Let Dr. Douglas answer to this. The present discrepancy of opinions, observes this physician, with regard to the nature of puerperal fever, whether it be an inflammatory, or putrid, or nervous disease, is attributable to a want of proper nosological distinction. Of the disease generally known under the name of puerperal fever, there are three distinct species. Dr. Douglas thus enumerates them:

Synochal puerperal fever,
Gastro-bilious puerperal fever,
Epidemical or contagious puerperal fever.

"The cases that I rank under the first distinction are all those attended with pyrexia, similar to that of any other purely inflammatory disease. These are neither produced by contagion, nor are they themselves infectious. * * *

"This form of the disease is to be subdued by copious blood-letting, and other sedative and evacuant remedies,—viz. antimonials, purgatives, enemata, fomentations, &c. The abstraction of blood, however, should here be considered our sheet-anchor: this fluid must not only be early and copiously taken, but the operation should be repeated in proportion as the pulse or abdominal pain may afterwards indicate. The pulse, I may remark, is often in this, as in every variety of abdominal inflammation, deceitful. I therefore regard the degree of pain felt on pressure as a preferable criterion, by which to regulate the abstraction of blood; and I cannot refrain from stating it to be my opinion, that the reason of this inestimable remedy having so frequently failed of the desired effect, and having been by some condemned as useless, or even injurious, in this and other diseases, is not owing to general inaptitude in the remedy, but to the inefficient manner in which it is often applied by timid practitioners." (*Id.* p. 152, 153.)

This, in fact, is no other than the puerperal peritonitis, to describe which, and the treatment thereof, Dr. Campbell has employed nearly the same language we have just quoted.

"Within the second distinction I include those cases wherein the disease does not so rapidly assume, or at least manifest, a decidedly inflammatory character: not, as in the former, commencing with a bounding, incompressible pulse; but with a pulse frequent, hard, and concentrated, as is usually observed in synochus, or the common epidemic fever: neither are the symptoms of abdominal inflammation so early evolved. Yet such inflammation does exist, and progresses when not checked, although more slowly and more obscurely than in the former. The tongue is here loaded, as in common bilious fever; whilst in the former it is usually white or cleanly florid, with sometimes a glazed appearance. The treatment here should likewise commence with blood-letting, but in more moderate quantity; immediately after which ten or twelve grains of calomel should be administered, and followed in a few hours by an ounce of castor-oil, combined with some other briskly-purgative medicine. These medicines, unless given in large doses, will produce but little effect in this complaint; and it is of paramount importance that the mucous surface of the intestinal canal should be effectually acted upon early in the disease in all cases, and more particularly whilst the morbid action of the peritoneal covering is under a kind of panic from the blood letting, and before that inflammatory action can re-organize its broken force. Often, by such prompt treatment, will the disease be arrested *in limine:* should it, however, advance, whether in increased or subdued violence, purgative medicines, varying in kind, must be daily administered, assisted by enemata, fomentations, topical blood-letting, &c. (*Id.* p. 153, 154.)

Who does not recognize in this statement the intestinal fever of lying-in women, first described by Dr. Granville, in his Report on Midwifery, under that appellation; long afterwards noticed by Dr. Hall, of Nottingham, under another name; and the existence of which, though hinted at by Burns, has never been as much as suspected by Armstrong, Gordon, Campbell, Macdonald, and others.

"That form of the disease which I arrange under the third head, is the really contagious or epidemical puerperal fever, and although agreeing with the others in the great leading symptoms, inflammation, pain, tumefaction, and tension of the abdomen, yet differing from them in many material characters. The sensorium here is seldom in any degree disturbed; whereas in the others it is so frequently, and even sometimes is excited to high delirium. The pulse here is usually, from the moment of attack, soft, weak, and yielding, and in quickness often exceeds 160; whereas in the first species it is full, bounding, and incompressible; and in the second, small, hard, and concentrated; and in both moderately quick. The eye, instead of being suffused with a reddish or yellow tint, as in the others, is here generally pellucid, with dilated pupil. The countenance, instead of being flushed as in the others, is here pale and shrunk, with an indescribable expression of anxiety; an expression altogether so peculiar, that the disease could, on many occasions, be pronounced or inferred from the countenance alone. The surface of the body, instead of being, as in the others, dry and of high pyrexial heat, is here usually soft and clammy, and of heat not above the natural temperature; and not only is the skin cool with clammy exudation, but the muscles, to the impression of the finger, feel soft and flaccid, as if deprived of their *vis insita* by the influence of the contagion. Indeed, there is such prostration of muscular strength and depression of vital principle, from the very onset of the attack, that I must suppose the contagion to act upon the human frame, probably through the medium of the nervous system, in a manner analogous to that of the contagion of the plague; and perhaps the African plague does not commit greater havoc among an equal number of infected persons than puerperal fever in this country: nor is puerperal fever less quickly fatal than the plague itself." (*Id.* p. 154—156.)

The treatment must vary like the disease. In the first species, bleeding freely and frequently, as recommended by almost every writer of note who preceded Dr. Campbell, must be relied upon for success. It is really too much to hear the Doctor declare that he thinks it his duty to come forward and inculcate the necessity of bleeding. He calls venesection "our sheet-anchor:" many years before him, the author of the Report on Midwifery (never once quoted or alluded to by the worthy Doctor,) gave venesection a stronger appellation, by calling it the "heroic remedy" in peritoneal inflammation of lying-in women. Dr. Douglas, we have just seen, urges the same remedy: so does every body. Is Dr. Campbell ignorant of this fact?

In the second variety, or intestinal fever of puerperal women, (the gastro-bilious of Douglas,) which, be it understood, is also accompanied by abdominal pain increased on pressure, bleeding, at least generally, is seldom necessary. If the pain has lasted some time before medical aid is summoned, topical bleeding may be required, and will be of service; but the principal means of relief must be sought in brisk and repeated purgation. We have seen cases of this variety so closely simulating the more simple variety of the disease (peritonitis), that nothing but previous experience could have restrained us from using the lancet. In the case of the lady of a physician, who was attacked with fever on the third day, accompanied by pain in the abdomen increased on pressure, and producing distention,—suppressed lochial discharge,—pulse above 100, and smart,—tongue brown, breath fetid,—lowness of spirits,—prostration of strength, —head-ache, &c. we might have done irreparable mischief had we been *exclusivists* with respect to the monopathology of childbed fevers accompanied with pain, and had bled the patient. We did no such thing. We purged freely with large doses of calomel at first, and kept up intestinal action by saline purgatives. The fever lasted a whole week: each free purgation brought relief, and the disease was subdued at last by the same means. There were at one time symptoms of putrescency; besides the offensiveness of the breath, of the lochial discharge, and of the alvine secretion, putrid effluvia exhaled from the body. The nitric acid was therefore ordered in a pleasant kind of beverage, and, we have reason for asserting, with most manifest advantage.

Of the *methodus medendi* in the third species of puerperal fever noticed by Dr. Douglas, we shall let that experienced practitioner speak for himself.

"Without pretending to detail any of the various modes of treatment which I may have seen pursued, either successfully or otherwise, I would here recommend the practitioner to commence by administering ten grains of calomel, combined with two grains of powdered opium, in the form of bolus or of pills; likewise, as early as possible, a briskly purgative enema. After the operation of the enema, a number of leeches, from two to four dozen, according to circumstances, should be applied to the abdomen, and the abdomen should afterwards be stuped with flannel clothes, wrung from warm water; and not only at this period, but frequently through the whole course of the disease, should such fomentations be used. Three or four hours having elapsed from the time of administering the calomel and opium, three drachms of pure oil of turpentine, with three drachms of syrup and six drachms of water, in the form of a draught, should be swallowed; and, after the lapse of another hour, this is to be followed by an ounce of castor-oil, or some other briskly-purgative medicine. In some instances, the oil of turpentine and castor-oil may be combined in one draught; but I generally prefer giving the turpentine as here recommended. Some of these remedies, occasionally assisted by others suitable to the peculiarities of the case, are to be repeated as circumstances may indicate; but I would not be disposed to repeat the internal use of turpentine oftener than twice, in any case whatever. In several cases, particularly where the debility is very considerable, the blood-letting may be altogether omitted; and in these cases a flannel cloth, sopped in oil of turpentine, should be applied to the abdomen, and allowed to remain on for the space of about fifteen minutes. This external application of turpentine, without either its internal use or the aid of blood-letting, I have frequently experienced to be entirely efficacious in curing puerperal attacks; and, although I have hitherto omitted to speak of turpentine for the cure of the other varieties of this diseases, yet I would not feel as if I were doing justice to the community, if I did not distinctly state that I consider it, *when judiciously administered*, more generally suitable, and more effectually remedial, than any other medicine yet proposed. I can safely aver I have seen women recover, apparently by its influence, from almost hopeless conditions;—certainly after every hope of recovery, under ordinary treatment, had been relinquished." (*Id.* p. 156—157.)

We have said nothing of Dr. Campbell's diagnosis and prognosis, as much of what has been observed under the head of symptomatology and pathology of the disease applies to the former, and no new facts respecting the latter have been brought forward by the author. As applied to simple peritonitis, his diagnosis is correct; but we cannot so far forget our own experience, or disregard that of Dr. Douglas and of many more, as to suppose that the symptoms Dr. Campbell has assigned to puerperal peritonitis, are not to be found in connexion with any other fever of childbed. That they are most of them present in intestinal fever, for example, in which bleeding is not "our sheet-anchor," we have proved *ad nauseam.* Ex uno disce omnes.

We have put ourselves nearly out of breath with this long discussion, and must hurry on to the conclusion of our observations on the very important question it involves, by saying a few words on the cases detailed by Dr. Campbell, and the success of his practice.

Dr. Campbell states that, from March, 1821, to September, 1822, a period of seventeen months, he and his assistants delivered 789 patients, of whom seventy-nine were affected with the disease he calls puerperal fever; of which latter number, twenty-two died. Here then we have a *prima facie* case of want of success; for the mortality amounts to within a fraction of ONE in THREE! If the seventy-nine patients were all attacked with simple peritonitis, as Dr. Campbell has written a thick octavo to prove, we contend that the mortality is far greater than usual. Not so, if many out of the seventy-nine patients were cases of either the second or third species of Dr. Douglas's puerperal fever, and bleeding was resorted to, "without any shameful timidity," for their cure. We have seen that Dr. Douglas rates the mortality in puerperal *peritonitis* as one in six only; and, by reference to the Report of Midwifery before alluded to, we find that, of twenty cases of *peritonitis post partum*, ONE only died. Tried, therefore, by the test of such comparisons, our author's practice cannot be considered as having been successful. We have shown, in a former article, how much more unsuccessful that of his colleague, Dr. Mackintosh, has been; and we need not go far to prove that the main cause of this want of success is to be found in those two gentlemen having classed together, and treated as one and the same complaint, affections that have been shown to be different by men of far greater experience than the authors in question.

We have already had occasion, in a note to the present article, to allude to the failure of Dr. Campbell's "sheet anchor" in saving many of his patients from "shipwreck;" and we have now further to remark, that, of the numerous cases reported by our author, those proved fatal in which the patients had been *most freely bled,*—as did some who had been so but sparingly. In some cases, indeed, we have venesection ordered and repeated, from the mere circumstance of pain in the abdomen being present; no mention being made of the state of the pulse at the time, nor of any other symptoms. In other cases, where bleeding had been practised most freely, the disease followed its course unchecked and unmodified by the remedy; till at last we find Dr. Campbell, for no reason that can appear, and notwithstanding the permanency of the abdominal pain, (his "unerring test of inflammation in this disease,") stopping short with it, and ordering brandy or some other cordial,—the patient dying notwithstanding.

We feel so keenly the importance of not suffering such books as Dr. Campbell's and Dr. Mackintosh's to mislead the yet-unformed practitioners in midwifery in their ideas, conceptions, management, and treatment, of fevers in child-bed, that, in spite of the great length to which the present article has already extended, we must enter more minutely into the consideration of the greater part of the cases brought forward in the present instance.

CASE I.—Delivered 26th March, 1821; attacked with fever on the 28th. Bled that evening to twenty-five ounces. On the 29th, much better. On the 30th, abdomen tumid and painful. VS. 18℥, at 10 A.M.; VS. 12℥, at 1 P.M. On the 31st, sufferings aggravated.—VS. 18℥. Dies April 2d. The pain and tumefaction of the abdomen not at all checked in their development by the bleeding. Total blood lost, seventy-three ounces. The case was promiscuously attended by Dr. Campbell, Dr. Murphy, and Mr. Lizars.

*Dissection.*—Intestines, mesentery, omentum, and uterus, said to exhibit marks of inflammation; the former glued together by coagulable lymph. Eight ounces of purulent fluid effused into the cavity of the abdomen.

☞ It is worthy of remark that Dr. Campbell gave four grains of calomel the first day of the attack; and that, because there existed diarrhoea after it, no more purgatives were administered throughout the disease, but the chalk-mixture instead. The day after taking the calomel the patient was much better.

CASE II.—Delivered by Dr. Murphy, April the 3d, the day on which the dissection of No. I. took place, at which it is inferred that the Doctor was present. Had been in labour three days, and was bled to eighteen ounces during it. Attacked with fever the same day, but not seen till the day after, when she was bled to twenty-six ounces. Dr. Campbell visited her in the afternoon. VS. repeated to twenty ounces; two doses of calomel. Cured. The appearances of the blood not mentioned. Total blood lost, 64℥.

CASE III.—Delivered, 4th April, by Mr. Young; attacked with peritonitis (vera) the following day. VS. 32℥; blood cupped and buffy. Two grains of calomel, twice, which produced several liquid stools. Cured. Total blood lost, thirty-two ounces.

CASE IV.—Delivered, 5th April, by Dr. Campbell. Attacked with fever early on the 6th of April: visited in the afternoon of the same day. VS. 32℥; two grains of calomel; VS. 18℥.—April 7th, VS. 12℥: in the evening free from pain, but, as a matter of precaution, VS. again to 18℥.—April 9th and 10th, nothing remarkable.—April 11th, pain returned, for which (says the author,) the patient could assign no reason: VS. ad f℥ 24, and 24 leeches. Three hours afterwards, VS. to f℥ 9. She had, some days afterwards, a severe attack of phlegmasia dolens, but ultimately recovered. She was attended by two other of the medical gentlemen who, with Dr. Campbell, were then visiting some of the cases already detailed. The communication thus kept up was unlimited. Total blood lost, 113 ounces: the appearances of the blood not hinted at.

CASE V.—Delivered by Dr. Campbell, the same day he bled the preceding case for puerperal fever, (7th April.) 8th. Rigors, with heat and acute pain in the lower part of the abdomen: VS. 18℥; calomel two grains, bis. On the 9th, bowels moved, free from pain. On the 10th, quite easy till ten P.M., when the patient was found in a state of great agony from abdominal pain: VS. 30℥. On the 11th, VS. 18℥; at one P.M. same day, VS. 24℥. On the 12th, she expired. Total blood lost, ninety ounces. No where is the appearance of the blood marked in this case.

*Dissection.*—Present Dr. Murphy and others. The uterus and its appendages were the parts which suffered most: peritoneal coat greatly inflamed; broad and round ligaments still more so. Ovaries greatly enlarged; the left in a state of suppuration. Effusion of serum small; intestines did not cohere.

CASE VI.—Attended by Dr. Campbell and Dr. Murphy; delivered April 8th. On the 14th, attacked with symptoms of fever: seen four hours after the rigors by Dr. Campbell. Abdomen tumid and sore. VS. ℥30. Cured.

CASE VII.—Delivered by one of Dr. Campbell's pupils, 19th April. On the 25th, head-ache; pulse 120, and strong; abdomen painful. VS. 18℥; at three P.M. VS. 14℥, and three grains of calomel, bis; at six P.M. VS. 13℥, and twenty-four leeches. April 26th, VS. 15℥, twenty-four leeches; at three P.M. VS. 7℥; at nine P.M. VS. 10℥. On the 27th, VS. to 7℥. On the 28th, VS. 7℥, at ten o'clock; VS. 12℥, at noon; at four, VS. 7℥. On the 29th and 30th, much better.—May 1st, three P.M. fresh attack, under which she expired. Total blood lost, 110 ounces: the appearances of the blood no where mentioned.

⁂ From the 26th of April to the 30th, the state of the bowels is not even hinted at; and, with the exception of a common enema, no purgative medicine appears, from the journal, to have been administered.

Dissection could not be obtained.

CASE VIII.—Delivered by Dr. Campbell, the 20th of April. On the 23d, symptoms of fever: venesection 19℥; at four o'clock, venesection 12℥. Cured. Total blood lost, thirty-one ounces: the appearance of the blood not stated.

CASE IX.—Delivered May 22d; symptoms of fever same day. On the 29th, VS. 26℥, blood cupped and buffy; at three P.M. VS. 9℥; at eight P.M. VS. 8℥; at 11 P.M. VS. 12℥. On the 25th, fifteen leeches. On the 26th, VS. 16℥. Cured. Total blood lost, seventy ounces.

CASE X.—Delivered 31st May; on the 3d June, rigors, &c. Had had no alvine evacuations since delivery: venesection 28℥, and two ounces of salts; blood cupped with a buffy coat. Cured.

CASE XI.—Delivered, on a Tuesday, after a long and protracted labour, in which two attempts were made to assist the birth of the child. During labour she was bled twice; first, to sixteen ounces; next, to eighteen ounces. The child died, thirty-six hours after birth, in convulsions. On Wednesday evening, attacked with fever: venesection to seventeen ounces; at thirty minutes past three o'clock, to ten ounces; at nine o'clock, to twelve ounces; and at three o'clock, to nine ounces. The pain of the abdomen excruciating at half-past nine, and she died at noon in great agony. Total blood lost, eighty-two ounces; the first blood detracted is said to have been buffy, &c. The only medicine employed appears to have been Epsom salts.

⁂ There is great confusion in the dates of this case. She is taken in labour on the 10th, but not delivered till the next day at nine P.M. This, we are told after, was on a Tuesday, and the woman continued well "until late on Wednesday evening." Was that the 12th? If so, why is the next day's account dated the 19th? Perhaps, by Wednesday, Dr. Campbell meant Wednesday in the succeeding week; so that the woman was seized with fever "*late in the evening*" of the ninth day after delivery, which would, of course, be the 19th. But here again we are puzzled by Dr. Campbell's inaccuracy; for he says that he visited her at *eleven o'clock in the morning* of the 19th, and found her labouring under all the symptoms of puerperal fever! We really hope that we can rely better on the accuracy of the other details of these cases, and that the various facts have been noted at the time with somewhat more precision.

Dissection could not be obtained.

Case XII.—Delivered 25th July; attacked with fever on the 30th. VS. 22℥; at three o'clock P.M. VS. 10℥. Frequent dejections now took place, and the patient got well. Total blood lost, thirty-two ounces.

Case XIII.—Delivered by Dr. Campbell, 29th July; attacked with rigors, &c. on the 3d August. She had had *no evacuation since delivery*. Venesection to the extent of twenty-eight ounces, and two ounces of salts. On the 4th, no evacuation yet: venesection, eighteen ounces, and one ounce of castor-oil. At last she had several stools, containing hardened fæces, and she was relieved immediately. Total blood lost, forty-six ounces.

Case XIV.—Delivered on the 10th of August; shivering and fever came on at two o'clock A.M. of the 12th. VS. 25℥; at six o'clock P.M. VS. 12℥. Cured. Total blood lost, thirty-seven ounces.

Case XV.—Delivered on the 12th September; rigors on the 16th. Venesection, twenty-eight ounces. Cured.

Case XVI.—Abortion, between the second and third month, on the 10th October; fever, with pains of the abdomen, rigors, &c. on the 12th. On the 14th, visited by Dr. Campbell: too late to bleed; eighteen leeches applied to the abdomen. Patient died at 11 P.M. on the 15th.

*Dissection.*—Peritoneal covering of the intestines and uterus exhibiting increased vascularity; serous effusion into the cavity of the abdomen to twenty ounces.

Case XVII.—Delivered October 14th; symptoms of fever on the 15th. Visited on the 16th: VS. 28℥; on the 17th, VS. 17℥. Died on the 18th. Total blood lost, forty-five ounces. The only medical agents employed in this case were "a saline purgative," and ten drops of the liquor opii sedativus!

*Dissection.*—"The appearances of inflammation were by no means so remarkable as in the last (fatal) case." Dr. Campbell, with much "naiveté," concludes his account of the dissection thus:—"In the present instance, the disease had nearly been subdued." Effusion not considerable.

Case XVIII.—Delivered with the forceps, October 16th, in the morning; in the night, symptoms of fever. Visited at eleven A.M. on the 17th: VS. 16℥. On the 18th, one ounce of castor-oil; pulse 88. On the 19th, she was visited by Dr. Campbell: venesection sixteen ounces, and twenty-four leeches. Up to the 20th, bowels constipated. In the evening, pain returned with great severity: twelve leeches applied to the abdomen, and a tobacco injection. On the 21st (Sunday), pulse 160; nothing done. On the 22d, no account. On the 23d, the patient died, late in the evening, in great pain. Total blood lost, thirty-two ounces. Neither in this nor in the preceding case is the appearance of the blood mentioned.

*Dissection.*—The vascularity of the peritoneum was not generally increased. Neither the uterus nor its appendages exhibited so much vascularity as the other parts. There were clots of coagulable lymph; and the effusion more considerable than in the last case.

Case XIX.—More want of precision as to dates. We take it, the woman was delivered by Dr. Campbell on the 22d October. He bled her during the labour to eighteen ounces, to facilitate delivery: this did not seem to have its full effect, in consequence of which the Doctor proposed to tie up the arm a second time. This being resisted, the forceps was substituted. On the 23d, restless, but no pain: castor oil, one ounce. At six o'clock P.M. no evacuation. October 24th, rigors; abdomen painful, pulse 112: VS. 42℥; blood buffy. Cured. The bowels here were well moved after the bleeding. Total blood lost, sixty ounces.

Case XX.—Delivered on the 22d October. During the labour, Dr. Campbell bled her to fifteen ounces, "on account of the condition of the passages." Two hours after the bleeding, "there was scarcely any advance of the head," when the forceps was used. On the 25th, she was visited by Dr.

Orr, who "found her labouring under all the symptoms of puerperal fever:" VS. 24℥. Cured that same day. Total blood lost, forty-nine ounces.

CASE XXI.—Delivered 26th October, having been bled during labour, (quantity not stated.) On the morning of the 29th, Dr. Campbell "found her in a condition highly characteristic of the first stage of this insidious disease." Venesection twenty ounces. Cured.

CASE XXII.—Delivered 31st October; symptoms of fever on the 1st of November. Visited on the 2d; abdomen tumid and painful: pulse 140, VS. 45℥. At half-past one o'clock, visited by Dr. Campbell: VS. 16℥. No evacuation of the bowels. *Neither of the detractions of blood exhibited the buffy coat.* At five o'clock, VS. 9℥, and four tobacco glysters in the evening. November 3d, VS. 10℥.; at one o'clock, VS. 7℥. The patient died on the 5th instant. Total blood lost, eighty-seven ounces.

CASE XXIII.—Delivered 31st October. On the third evening, symptoms of high excitement, particularly in the head: VS. 26℥; at two o'clock P.M. VS. 16℥. Both detractions of blood buffy. Bowels still constipated: in the evening, enemata were administered; but they produced little effect. VS. 10℥. November 5th, several copious liquid stools. November 6th, VS. 16℥. At two P.M. of the same day, pulse 140: VS. 10℥. After this, maniacal symptoms. Dr. Campbell gave up visiting her on the 16th of November, when the symptoms of mania had considerably subsided; but he understood afterwards that she was labouring under symptoms of phthisis. Total blood lost, seventy-eight ounces.

CASE XXIV.—Abortion before the fourth month, on the 2d of November. On the 4th, rigors, (she was examined per vaginam both by Dr. Campbell and Dr. Orr, who had attended the three or four preceding cases, nearly on the same day,) and other symptoms of fever: VS. 24℥. At eight o'clock P.M. abdomen as tender as at the last visit. The blood detracted did not appear sizy; VS. 16℥; no stools. November 5th, four alvine evacuations during the night; abdomen free from pain. Cured. Total blood lost, forty ounces.

We have now given as much of the evidence brought forward by Dr. Campbell, in support of his opinion respecting the nature and treatment of what he has called "puerperal fever," as will enable our readers to judge of the correctness of our critical remarks on the subject of both. Of forty-eight cases related by our author, we have analysed the first twenty-four in succession, and consequently without any attempt at selection. Of the remaining four-and-twenty cases, we shall only give the total quantity of blood detracted in their treatment.

The conclusions which naturally suggest themselves on the contemplation of the evidence laid before us, may be classed under three distinct heads. The first have a reference to Dr. Campbell's nosological discrimination. The second to his practice, and mode of detailing the case. The third to his success.

1. That Dr. Campbell has designated by one and the same name, and considered as one and the same disease, different puerperal affections attended with fever, there can be no doubt. We admit that he has seen a great proportion of cases of simple puerperal peritonitis, in which bleeding has been of the greatest service. But we must also freely say, that he has bled (and very largely too,) where no success from that measure was to be expected; and where, consequently, no success followed, because the disease was not simple puerperal peritonitis. We appeal to those of our readers who have had most practice in these complaints, whether, in the enumeration of Dr. Campbell's patients, they do not find some that were affected with the *true*, or contagious puerperal fever of Dr. Douglas; others, whose complaint was, in the first instance, intestinal fever, requiring purging, and not bleeding; and not a few labouring under the peculiar affection well described by Dr. Hall, consisting in nervous irritation or exhaustion. We have also two or three instances of decided *mania puerperarum:* and yet all these various diseases are set down as *peritonitis puerperarum*,—are grouped under the same designation in Dr. Campbell's volume, —and are to be treated, according to him, in one and the same manner, viz. by venesection and leeches.

Will Dr. Campbell deny that any difference exists between the symptoms in Case I. and III., for instance? What are the two fatal cases, V. and VII., but examples of the typhoid puerperal fever? Is Case X. any thing like that which follows it? Or is Case XII. like either?

Under this head, therefore, our conclusion must be, that Dr. Campbell's nosological discrimination in puerperal fevers is deficient.

II. If Dr. Campbell's diagnosis appears to have been erroneous, it follows that his practice must have been alike in fault. We find, in fact, that such was the ill-fated determination to see nothing but puerperal fever in those of the women delivered by him or his assistants, who had rigors, head-ache, and soreness of the abdomen, that not a word is to be found in the narration implying that a lying-in woman might be affected by any other complaint. A woman (Case XIII.) had no alvine evacuation for six days after delivery; and Dr. Campbell first bleeds her, and then gives her two ounces of Epsom salts, which produce no effect. Can our author be serious when he relates this as a case of genuine peritonitis?

In Cases XVI. XVII. and XVIII. which proved fatal in spite of the lancet, no attempt was made to subdue the disease by any other means, although it was evident that the bleeding did not produce the desired effect. Not even the fraction of a grain of calomel was administered to the patients! But, even with regard to his exclusive remedy, venesection, Dr. Campbell is so inconsistent, that it would be very unsafe to follow him as a guide in these matters. In one case, presenting symptoms by no means alarming, thirty and forty ounces of blood are directed to be taken at once. In another, where the fever and the state of the head and pulse are such as to threaten much worse consequences, sixteen ounces only are abstracted. Dr. Campbell lost several patients whom he had bled repeatedly, and, as we have shown in another part of this article, to a considerable amount: but he has also lost some where only one or two small bleedings had been practised. We profess not to be able to trace the reason for such distinctions. In Case VII., which proved fatal, ten different bleedings were ordered in the course of four days. But in Case XVIII. five days were suffered to elapse with two bleedings only, each of sixteen ounces. We do not mean to decide whether bleeding was proper or improper in either case; but we mention these two cases, out of many more, to contrast Dr. Campbell's practice in two examples of what he himself considered the same disease.

In Case XVIII. relief was at hand at an early period of the disease, the first attack having come on during the night previous to the visit of the physician; yet the bold practice of bleeding, as followed in Case XXII. for instance, where forty-five ounces were taken from the arm at one time, was not adopted; the reason of which we cannot discover. Neither were any other remedial means resorted to in this case. On the day she was bled, no opening medicine was given. On the day following, an ounce of castor-oil was directed to be taken; but we are not told with what effect. Dr. Campbell saw the patient for the first time the day after, and he ordered a second bleeding of sixteen ounces, and an enema. Another day elapses, and we are told that the bowels were still obstinately constipated. At last a second ounce of castor-oil was administered, which produced two evacuations, and consequent relief. We are informed, however, that she had a relapse in the evening. Well might she;—in intestinal fever, (and the present is one of the strongest instances we have read,) relapses may invariably be expected where only two motions are produced in five days; and those by the administration of an enema and two ounces of castor-oil only. The relapse was characterised by a pulse at 130, and pain of the abdomen returning with increased severity. Why then, in the name of consistency, if Dr. Campbell was right in his view of the case, (peritonitis,) and thought he had acted correctly in similar cases, where slighter relapses were met with copious venesection, did he not have recourse to his "sheet anchor" to save the patient, instead of the mere application of twelve leeches to the abdomen. But, in our opinion, purgatives, especially calomel, jalap, and cream of tartar, ought to have been used from the beginning, with frequent enemata; and these ought to have been employed even on the relapse taking place, instead of the tobacco-injection which could only have been tried as an experiment. The next day the usual train of symptoms happened, which accompanies to their fatal termination all such cases of intestinal fever as have been suffered to proceed so far as to produce inflammation. Prostration of strength,—pulse 120, and thready,—vomiting,—liquid and offensive stools,—tongue and teeth encrusted,—death. For three days previous to this, nothing appears to have been done subsequent to the application of the twelve leeches and the tobacco-injection.

The preceding case speaks more for Dr. Campbell's great candour, than for his nosological discrimination and activity of practice; yet he has shown that he can be bold enough with the lancet in most cases.

We shall mention another case of Dr. Campbell's "puerperal fever," taken from among those patients who recovered. We might comment on many more, but we feel we have trespassed far enough on the time and patience of our readers. The case is marked No. XIX. The woman was in labour of her first child, and was bled to the extent of eighteen ounces, for no other purpose, that one can guess, except that of accelerating the dilatation of the passages, as the author observes; though it is acknowledged that the labour, which had only lasted twelve or fourteen hours from the very first indication of it, "was well advanced." The head of the fœtus got at last to the perineum; and here it remained many hours. Dr. Campbell now proposed further bleeding; but, on his proposition being resisted by the patient and her friends, the use of the forceps was suggested as a substitute, adopted, and found useful. We are not told what was done for the patient this day. On the following day we find the patient restless, with *no local pain*, pulse 96. An ounce of castor-oil was given. At six in the evening of the same day, the bowels were still unmoved. Throbbing sensation in the abdomen,—pulse between 90 and 100: an injection. On the fourth day, patient restless; had a slight rigor in the morning; a slight pain darting occasionally from the lower part of the body to the left side, by no means troublesome. Could not bear pressure to any extent. How could she, indeed? The "purgative medicines had not been attended with the desired effect." The fourth day, and no passage! Well may the pulse be at 112. Dr. Campbell, having now detected peritonitis by his test of pressure on the abdomen, bled the patient at once to forty-two ounces. (We have seen that in Case XVIII., where the symptoms appear by the description to have been much more alarming, only *sixteen* ounces of blood had been abstracted.) On reading this, we felt rather uneasy as to the fate of the patient. Luckily an ounce and a half of Epsom salts were administered at the same time, and produced three copious liquid, frothy, bilious stools, succeeded by several more before eight o'clock of the same day. From that moment we looked upon the woman as safe. The abdomen became "perfectly easy." "She felt (indeed) exceedingly faint from the loss of blood." She had also "rather a lingering convalescence:" all natural consequences, and to be expected; but she got well at last.

With regard to Dr. Campbell's mode of relating his cases, there are some deficiencies and omissions, the occurrence of which take much from the clearness and precision of the detail. The number of pulsations is frequently given without stating the nature of the pulse. With some few exceptions, the appearances of the blood after venesection are never hinted at. The condition of the bowels, even after announcing an attack of fever several days after labour, is wholly overlooked; and, except in one or two cases, the nature of the evacuation is never alluded to. Dr. Campbell is equally negligent with regard to several important points in the narration of his post-mortem examinations. The cavities of the head and thorax were never inspected, or at least are not mentioned: the same remark applies to the state of the mucous membrane of the stomach and bowels. The internal surface of the uterus was seldom examined. The quantity of serum effused is loosely guessed at, instead of being carefully measured; especially as Dr. Campbell has often compared the effusion in the one case to that in another, of neither of which the precise quantity is given.

As to our conclusions under the third head, namely, that which has reference to Dr. Campbell's success, we shall be equally impartial, but more brief. Indeed, we have already been pretty explicit in this respect. Dr. Campbell has seen seventy-nine cases of fever after parturition, of which he lost twenty-two. He has detailed forty-eight cases, in the treatment of which he has taken away TWO THOUSAND TWO HUNDRED AND THIRTY-FOUR OUNCES OF BLOOD, and applied eight hundred and sixty-two leeches; and it appears that sixteen of them proved fatal. If the seventy-nine cases in question were cases of genuine peritonitis puerperarum, as Dr. Campbell thinks, then he has been more unsuccessful than his brethren, using the lancet more extensively than they do; for he has lost nearly one patient in three. If, on the contrary, they be considered mixed cases of peritonitis, true puerperal fever, intestinal fever, &c. then Dr. Campbell has been equally unsuccessful with his contemporaries, and we do not see that he has any claim either to novelty or to superiority of practice.

In conclusion, we would desire to consider Dr. Campbell's book as containing several useful facts, and more than one instructive lesson to the junior practitioners in obstetric medicine.

—We may at some future period state more fully our own sentiments on the very important subject which has occupied so large a space in the present Number.

---

### DIVISION II.

### FOREIGN.

ART. III.—*Considerations Anatomiques, Physiologiques, et Pathologiques, sur la Luette.* Par le Docteur J. LISFRANC, Membre titulaire de l'Academie Royale de Medecine, Chirurgien du Bureau central d'Admission des Malades dans les Hôpitaux et Hospices civil de Paris, &c. &c.—(*Revue Medicale,* Juillet.)

THE object of M. LISFRANC in this Essay is to call the attention of practitioners, in a more especial manner than has hitherto been done, to the structure and functions of the uvula. The dimensions of this musculo-membranous prolongation, as the author denominates it, are extremely variable in different individuals: some instances are recorded in which it has consisted merely of a short tubercle; and one is mentioned by M. Lisfranc, in which it was of the ordinary length, but as fine as a thread; yet in neither case was the voice at all altered. In the "Ephemerides des Curieux de la Nature," an instance is mentioned of a girl, who had been deprived of the uvula from the period of birth, and yet no change of voice took place. In other examples this organ has been bifid, without the existence of hare-lip or division of the palate. The uvula is wanting in all the lower animals, although the rudiments may be seen in some, particularly of the monkey tribe.

What are the functions of this organ? Does it assist in the formation of the voice, and the articulation of sound? This idea would seem to be contradicted by the many instances in which the uvula has been removed without the voice being affected; and a case is related by the author, which appears conclusive on this subject. "It is well known (says he,) to many medical men, that venereal sores destroyed the epiglottis, the pillars of the arch of the palate, and the uvula, in one of the most distinguished performers on the lyric scene, and that his voice lost nothing in the flexibility or clearness of its tone." The author agrees with M. Richerand, that the uvula serves to give notice to the pharynx of the arrival of food; but that is not its sole office. He continues: "When we wish to transmit to the pharynx the humours secreted by the schnederian membrane, we close the mouth, we open the anterior fauces, and introduce the largest possible quantity of air into the nasal cavity; the air must pass through this part with difficulty, in order that it may be applied to its sides with greater force, and clear them more effectually. In order to effect this, the arch of the palate is carried upwards and forwards; the uvula in the natural state follows this movement: situated on the median line, like the larynx, it forms a kind of projection to the right and left of the foreign bodies, which would fall into the cavity of that organ. This musculo-membranous part of the arch of the palate serves, therefore, the additional purpose of preventing the mucus of the nostrils from penetrating into the larynx: nature, besides, has disposed the arch of the palate in such a manner as to form two lateral gutters."

Various proofs are advanced in illustration of this. First, it is only necessary to place ourselves before a looking-glass, and to open the mouth, taking in a deep breath at the same time, in order to see the arch of the palate and the uvula perform the movements here indicated. Secondly, the rudiment of this organ possessed by different animals is proportionally more distinct as they have the head less frequently inclined towards the earth, and as they approach the human species: thus, in the red orang-outang, the organization of which most resembles ours, the uvula is almost complete; while in some kinds of monkey, of remote resemblance to the human species, scarcely any traces of it are to be found. Besides, in those animals whose conformation gives ready vent to the nasal secretions, the arch of the palate extends still farther into the pharynx than in man. Thirdly, it is observed by the author, that, when the uvula is in a state of complete relaxation, and when it has been entirely removed, the mucus from the nares falling into the posterior fauces is apt to get into the glottis, unless the respiration is performed with considerable slowness and precaution.

It sometimes happens that the uvula remains in a state of undue relaxation, as a consequence of a violent quinsey, resisting the usual application of astringent gargles and similar remedies. Among other things, the author recommends the application of pepper or powdered ginger, carried to the part in a small spoon. We have been informed by a surgeon of experience, that he has frequently succeeded in removing this relaxed state by touching the part with a hair-pencil dipped in the tincture of capsicum; a method certainly of much more ready application than that of M. Lisfranc. Various inconveniences are apt to arise from elongation of the uvula,—such as attacks of inflammation; irritation giving a constant tendency to swallow, and sometimes producing a frequent cough. This last effect has been particularly noticed by Dr. Physick in America, as being mistaken for phthisis pulmonalis, (a circumstance which we mentioned in the "Intelligence" of a former Number;) and we have had occasion ourselves to know of a case of this kind, which was treated for several months as consumption, and ultimately cured by an operation.* This, indeed, is frequently not merely the best, but the only means, by which the evil can be remedied. With respect to the manner in which this may be best effected, M. Lisfranc speaks as follows:

"Almost all authors advise us only to remove half of the uvula, when its diseased state does not extend too far: I have observed that, under this plan, the part swells and becomes elongated anew, so as almost always to require excision a second time. I believe, after a great many observations, that it is preferable to remove it completely at once. We have already shown that the voice and speech experience no change: it is, then, erroneously asserted, in the "Dictionnaire des Sciences Medicales," that the uvula concurs in the formation of certain sounds, particularly of the letter R, and that articulation is lost when this part is removed. All physicians are convinced that Aphrodiseus abused the public confidence, when, under Antoninus and Severus, he rendered himself famous by endeavouring to inspire a dread of fatal phthisis following as a consequence of extirpating the uvula at its root. Experience has proved to me that the operation, thus practised, has only the slight inconvenience of allowing the nasal mucus to fall more readily into the larynx: now this cannot be put into the scale against the risk of a second operation.

"Many methods of performing this operation have been proposed; we shall briefly relate them. Hippocrates says, that it is necessary to turn up the uvula with the finger, and cut off the point. Celsus seized the organ with a forceps, and removed *as much as he thought necessary.* Fabricius ab Aquapendente preferred the scissars alone: he says, that one hand is thus at liberty, and that he is thus able to manage the tongue and lower jaw. This author applied a spoon heated, but not incandescent, to the wound, in order to close the tissues, and perhaps to stop the bleeding. When the size of the uvula gives reason to apprehend this occurrence, Paré prefers the ligature. * * * Arnaud asserts, that in some cases the uvula becomes so hard as to resist the scissars and the ligature; while he adds, that the bistoury would be attended with danger. He contrived an instrument, consisting of a blade and sheath, the extremity of the latter being pierced with a circular aperture, for the purpose of engaging the uvula perpendicularly; when it is introduced, the instrument touches and lifts up the arch of the palate; the thumb is then placed on the handle of the blade, which is to be pressed with some force, and the organ is thus divided at a single incision. Heister has given an engraving of an instrument invented by a Norwegian peasant. Read improved this: it is composed of two sides, united at one end by a transverse part; their corresponding faces are grooved, so as to admit a knife which glides between them; the portion to be removed is fixed between the sides of the instrument, at its extremity, and the blade pushed upon it.

* See also our "INTELLIGENCE" for the present Number.

"Sabatier made use of a small polypus forceps, bent as scissars. Some have employed these bent into a semicircle, that the uvula might not slip from them; while others have invented straight scissars, having a narrow guard attached to the point of one of the blades. * *

"The ligature has the great inconvenience of giving intolerable pain, and frequently exciting violent inflammation, and is now abandoned. In the only example in which I have seen it used, the patient could not bear it. The instrument of Arnaud, and that of the peasant of Norway, are means which modern surgery no longer employs. If the organ be much indurated, it may be cut with the large scissars of M. Dubois. A cartilaginous uvula, which fell under my observation, did not resist these. * * * Whatever scissars be employed, the point must be blunt; those having the blades curved render it more difficult for the body placed between them to escape, and therefore they deserve the preference; and we think, besides, with Sabatier, that the part ought to be laid hold of with a small forceps. Notwithstanding this precaution, however, it sometimes happens that the organ, if excessively soft, is not cut through at one stroke. This arises from the root of the tongue preventing the instrument from being carried sufficiently far back. I propose, after having pulled the uvula forward, to turn it towards the right side of the mouth: thus the scissars may be placed almost transversely, and the diseased part will be engaged in the instrument almost at its joint, and it is impossible but that it must be immediately and completely removed. This plan has always succeeded with me. In some cases excision of the uvula is not painful, but in others the patients suffer a great deal. * * * When the uvula has been removed by operation, the surgeon must guard against inflammation; for which purpose astringents or emollients are to be employed, according to the indications. These attacks are generally too much neglected after the operation, and it is not astonishing to find hoarseness of the voice sometimes remaining permanent."*

### MEDICAL AND PHYSICAL INTELLIGENCE.

* See page 519, of this Number.

